# Perspectives for the Use of Neurotechnologies in Conjunction With Muscle Autotransplantation in Children

**DOI:** 10.3389/fnins.2019.00099

**Published:** 2019-02-15

**Authors:** Evgueni Blagovechtchenski, Olga Agranovich, Yelisaveta Kononova, Maria Nazarova, Vadim V. Nikulin

**Affiliations:** ^1^Centre for Cognition and Decision Making, Institute for Cognitive Neuroscience, National Research University Higher School of Economics, Moscow, Russia; ^2^The Turner Scientific Research Institute for Children's Orthopedics, Saint Petersburg, Russia; ^3^Federal Center for Cerebrovascular Pathology and Stroke, Moscow, Russia; ^4^Department of Neurology, Max Planck Institute for Human Cognitive and Brain Sciences, Leipzig, Germany

**Keywords:** neurotechnologies, autotransplantation, motor control, arthrogryposis, rehabilitation, neuroprosthesis

Muscles autotransplantation is an important way to restore motor activity in case of injury or diseases associated with a loss of muscles ability. One of the typical examples of such pathology is arthrogryposis multiplex congenita (AMC). Arthrogryposis is one of the most serious congenital malformations of the musculoskeletal system. It is characterized by the presence of two or more major joint contractures, muscle damage, and motoneuronal dysfunction in the anterior horns of the spinal cord. One of the main problems that determines the limitation or even impossibility of self-care of patients suffering from arthrogryposis is the lack of active movements in the upper limb joints, which can be restored by autotransplantation of the muscles of various donor areas (Hall, 1997; Bamshad et al., 2009; Loeffler and Lewis, 2016).

A major limiting factor for the adequate self-care in patients with this pathology is a lack of the active elbow flexion due to the fibro-fatty degeneration of the flexors of the forearm. Such deficits significantly affect the quality of life because many vital functions are associated with the elbow movements, for example, bringing food to the mouth. Thus, for these patients it is important to secure functional recovery of the biceps brachii muscle, which is performed by non-free (with preservation of the vascular-muscular bundle) autotransplantation of the muscles surrounding the shoulder joint (commonly by the pectoralis major or the latissimus dorsi muscles) (Oishi et al., 2017). The loss of the muscle function in the donor region does not cause any significant functional impairment due to the work of the remaining synergistic muscles (Mikati, 2007; Zargarbashi et al., 2017).

There are two pivotal and non-trivial aspects witch should be addressed for such surgeries:

1. Which muscle is the most suitable for the autotransplantation?
2. How to facilitate the rehabilitation processes after the muscle autotransplantation?

Next, we discuss these two issues in more detail.

## Which Muscle is the Most Suitable for the Autotransplantation?

At the first consideration of such a question, the most important criteria may be the anatomical, biomechanical, biochemical compatibility of the transplanted muscle to the original one (Hoang et al., 2018) and the peculiarities of their representations at the central nervous system level. The current criteria for choosing a donor muscle are: muscle strength of at least 3 points on a 5-point scale (MRC Scale for Muscle Strength) and the minimal damage to the donor area.

In this opinion we would like to focus primarily on the processes associated with the cortical muscles representations. Upper limb is used mainly to perform voluntary movements (unlike lower limbs whose most crucial function is locomotion), thus the cortical level plays here a crucial role (Pettersson et al., 2007; Lemon, 2008). The most studied question in this regard is the representation of the muscles in the cerebral cortex, i.e., motor homunculus, where the most pertinent question is whether cortical muscles' representations do exist at all or only synergies and movement characteristics are encoded in the cortex (Schieber, 2001). There is also a considerable difference in the amount of studies of distal and proximal muscles with very few studies addressing the question of the somatotopical interaction of the proximal muscles cortical representations (Kocak et al., 2009; Kesar et al., 2018).

Most readers are likely to be familiar with the picture from textbooks on how the motor homunculus is presented in the brain (Kocak et al., 2009) and there is a lot of data on the brain's plasticity (Nobre, 2001; Nazarova and Blagovechtchenski, 2015). It is sufficient to mention the so-called “mirror system” effect, where the excitability thresholds for TMS of the individual muscle vary depending on the observed movements, in order to emphasize how motor maps can dynamically change their presentations in the cortex (Buccino et al., 2004). Also of note is that the direct connection of the cortical neurons with the spinal motor neurons does not necessarily mean direct activation effect: the activity of the upper motor neuron does not always uniquely correlate with the activity of the lower motor neuron to which it is projected (Lemon et al., 1998; Lemon, 2008; Nazarova and Blagovechtchenski, 2015).

To our knowledge, it has not yet been investigated how cortical representation of a particular muscle can be associated with the possibility of its functional reconstruction in situation when the biomechanical position of this muscle is changed. Indeed, there is always a chance for the pathological plasticity leading to irrelevant movements and/or pain. It is generally known that some muscles have smaller cortical representation, and accordingly, less cortical voluntary control. Also we showed recently that there is a change in the basic EEG rhythms in children with arthrogryposis (Blagoveschenskiy et al., 2018), which can also be a reflection of the plastic changes in the motor cortex. Perhaps functional mapping of the cortical representations of the possible donor's muscles may give a better answer to the question–which of the donor muscles is most suitable for such an operation. How can one estimate the plasticity of a motor representation? We believe that the combination of non-invasive neuroimaging and stimulation approaches may allow exploring this issue more systematically. The degree of the involvement of cortical motor representations during muscle contraction can be assessed using MEG, EGG, and fMRI (Hopfinger et al., 2001). Fundamental in this case is the change in neuronal activity as a result of short-term motor learning. At the same time, it is necessary to estimate the corticospinal efficacy for such central rearrangements using a non-invasive stimulation approach, such as TMS.

## How to Facilitate Recovery and Rehabilitation Processes After Autotransplantation?

After muscle autotransplantation, many peripheral and central changes occur affecting all receptors and neurons associated with the movement process which is modified by surgery. One of the most interesting aspects of this problem is how to “explain” to the brain how to deal with those new degrees of freedom and control—i.e., elbow flexion—for which the brain had no control before.

A contemporary understanding of the movement organization includes such basic elements as the solution of an inverse problem, the formation of an efferent copy of the movement, which allow the brain comparing the planned motor pattern with the results of the performed action (Gallivan et al., 2018). However, in patients with arthrogryposis, there are no formed sensorimotor pattern associated with elbow flexion, or processing of the corresponding afferent signals and their comparison with an efferent copy. When a muscle is transplanted, the situation becomes even more complicated since the muscles get new biomechanical positions.

**Figure 1:**
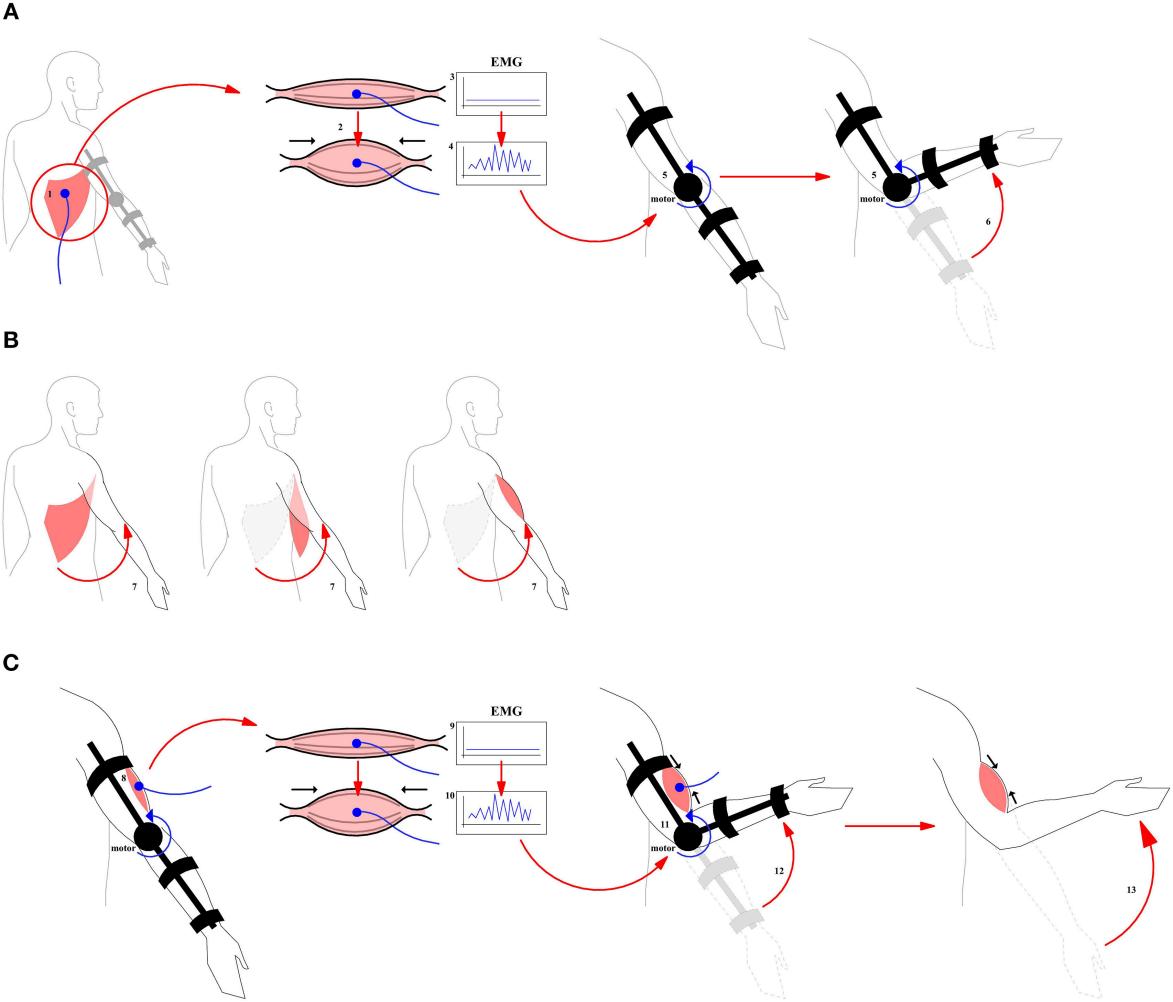
A general scheme for the use of prosthesis in the course of pre- and postoperative training. **(A)** Pre-operative training. A prosthesis is controlled by the EMG originating from the contraction of the donor muscle. (1) Donor muscle (2) Contraction of the donor muscle (3) EMG at baseline period (4) Control Signal (5) Prosthesis (6) Flexion produced by prosthesis. **(B)** Surgical intervention (7) Muscle transplantation. The muscle and its nerves are transplanted from their original location to a new location to perform a function of biceps brachii. **(C)** Post-operative training. A motor rehabilitation continues with a donor muscle being in a new place with the assistance of the prosthesis. (8) Donor muscle in the target position (9) New EMG baseline (10) Control Signal (11, 12) Flexion with the donor muscle plus assistance from the prosthesis (13) Flexion produced by the donor muscle without the assistance from the prosthesis.

In order to solve these problems, we propose to use not only postoperative rehabilitation but also a preoperative training aimed at the formation of central commands leading to the consolidation of new neurobiomechanical patterns functionally associated with the activation of a donor muscle. For example, let us consider a case of the donor muscle being latissimus dorsi, the activation of which is normally does not lead to elbow flexion. We propose to ask a patient to perform the contraction of this muscle even before the operation, which, of course, at this stage does not yet results in the flexion of the arm (Figure 1). However, we can use the electromyogram from the contracted muscle as a control signal to trigger a prosthesis that performs a mechanical elbow flexion. Thus, we do not require the patient to exercise their own flexion, we rather hypothesize that as a result of this training, there would be an association between the formation of the central motor command for the mechanical elbow flexion and the contraction of the future donor muscle–latissimus dorsi muscle in this case (see Figure 1). After the surgery, when the latissimus dorsi muscle is in the position of the bicep brachii, the patient is asked again to execute a contraction of latissimus dorsi which for the first time would be associated with the elbow flexion. Initially after the recovery, these attempts will have an assistance of the prosthesis which later can be removed.

At the present moment it is not known what may be the effect of the described preoperative training on the speed of the rehabilitation. Developing this new approach would require creating a simple prosthesis that performs an elbow flexion depending on the activity of the donor's muscles, which will be placed at the biceps brachii position. Importantly, already before the surgical intervention, an association should be formed in the brain between the contraction of the donor's muscle and the elbow flexion. Practical implementations would include the creation of a simple exoskeleton robot which controls the flexion of the arm in the special joint (in this case, the elbow), depending on the electromyogram of the muscle selected for the transplantation. In addition, in the initial pre-operative period, such robot-exoskeleton will control proper flexion, until the neuromotor patterns are stabilized. According to our observation, patients after the surgery prefer to contract the muscle isometrically, without changing the joint angle. We believe that using the described approach, it would be possible to avoid the dominance of the central descending commands which do not result in a flexor movement, since the electromyogram of the donor muscle will be used to perform the flexion. In a future prosthesis, the flexion would be launched only when a certain pattern on an EMG is reached that differs from the pattern corresponding to a simple isometric movement.

In conclusion, we suggest that:

1. The study of the cortical representations in the central nervous system of the muscles before their transplantation to a new position may play an important role in selecting the donor muscles.
2. Pre-operative training of the new biomechanical synergies based on the EMG activity from the donor muscle will allow speeding up the rehabilitation process.

## Author Contributions

All authors listed have made a substantial, direct and intellectual contribution to the work, and approved it for publication.

### Conflict of Interest Statement

The authors declare that the research was conducted in the absence of any commercial or financial relationships that could be construed as a potential conflict of interest.
